# Prevalence of Nutritional Deficiencies Among Patients Attending a Nutrition Clinic at a Tertiary Care Hospital in Andhra Pradesh, South India: A Retrospective Cross-Sectional Study

**DOI:** 10.7759/cureus.109115

**Published:** 2026-05-18

**Authors:** Arti Gupta, Vignesh D., Dhrubajyoti J Debnath, VenkatashivaReddy B., Rajeev Aravindakshan

**Affiliations:** 1 Community and Family Medicine, All India Institute of Medical Sciences, Mangalagiri, IND

**Keywords:** anemia, centre for nutritional health, family medicine, india, nutritional deficiencies, obesity, primary care, vitamin b12 deficiency, vitamin d deficiency

## Abstract

Background

Nutritional deficiencies persist as a major public health challenge in India alongside a rising non-communicable disease burden. The Center for Nutritional Health at AIIMS Mangalagiri provides comprehensive nutritional services including counseling, anthropometric assessment, and laboratory diagnosis of key deficiencies. We aimed to determine the prevalence of anemia, vitamin D and vitamin B12 deficiencies, and malnutrition (underweight and overweight/obesity); examine gender-based differences in nutritional parameters; identify patterns of multiple concurrent deficiencies; and analyze their age-wise distribution among patients attending the center, to inform targeted intervention strategies.

Methodology

We conducted a cross-sectional study using retrospective clinical data from 2,303 patients attending the Center for Nutritional Health at AIIMS Mangalagiri over 30 months. Demographic, anthropometric, hemoglobin, 25-hydroxyvitamin D (25(OH)D), vitamin B12, calcium, and 24-hour dietary recall data were obtained from routine clinical records. Deficiencies were defined using World Health Organization (WHO) anemia criteria, standard international vitamin reference ranges, and Asian-specific body mass index (BMI) cutoffs. Analyses included Pearson chi-square, Mann-Whitney U, Spearman rank correlation, and multivariable binary logistic regression.

Results

Among 2,303 participants (1,681 women (73.0%); mean age, 42.4 ± 13.9 years), anemia affected 1,195 of those tested (74.2%), of whom 764 (47.5%) had moderate-to-severe anemia. Vitamin D deficiency (<20 ng/mL) was present in 658 participants (59.5%), vitamin B12 deficiency in 250 (41.9%), overweight/obesity in 1,240 (78.0%), and underweight in 88 (5.5%). Women had higher odds of anemia (OR, 3.64; 95% CI, 2.83-4.68; *P* < 0.001) and suboptimal vitamin D status (insufficient/deficient, <30 ng/mL vs. sufficient, ≥30 ng/mL) (OR, 2.11; 95% CI, 1.17-3.81; *P* = 0.018). Multivariable regression identified female sex (adjusted OR, 1.60; 95% CI, 1.16-2.21; *P* = 0.004) and younger age (adjusted OR, 0.98 per year; 95% CI, 0.97-0.99; *P* = 0.001) as independent predictors of vitamin D deficiency (<20 ng/mL). Multiple concurrent deficiencies occurred in 386 (21.4%) patients, most commonly anemia with vitamin D deficiency in 182 (10.1%), with a female-to-male ratio of 3.1:1. Anemia peaked at ages <18 years 52 (81.2%) and 30-44 years 524 (80%); overweight/obesity was highest at 45-59 years, 395 (84.9%) and 30-44 years, 516 (82%).

Conclusions

This study documents a substantial burden of anemia, vitamin D and B12 deficiencies, and overweight/obesity among patients at a specialized primary-care nutrition center in South India, with women aged 30-44 years carrying the peak micronutrient-deficiency burden and adults aged 18-29 years representing the critical obesity-prevention window. Four key primary-care actions are needed: replacing indication-driven testing with a bundled baseline panel that includes hemoglobin, 25(OH)D, vitamin B12, and an Asian-specific BMI cutoff; prioritizing women aged 30 to 44 years for protocolized iron-folic acid and vitamin B12 supplementation with serum-guided vitamin D therapy instead of empirical treatment; providing structured lifestyle counseling to adults aged 18 to 29 years; and expanding food fortification efforts while integrating the center model into Ayushman Bharat Health and Wellness Centers to embed comprehensive nutritional care into routine primary care.

## Introduction

Nutritional deficiencies constitute a major global public health concern, with developing countries bearing a disproportionate burden [1]. India, despite rapid economic growth and an ongoing nutritional transition, continues to face a significant prevalence of micronutrient deficiencies alongside rising rates of obesity and non-communicable diseases [2,3]. This phenomenon, known as the *double burden of malnutrition*, poses unique challenges for healthcare systems and demands comprehensive assessment strategies in clinical settings [4].

Anemia remains one of the most prevalent nutritional disorders in India, affecting nearly half of women of reproductive age according to the National Family Health Survey-5 (NFHS-5) [5], with significant adverse effects on cognitive development, work productivity, and maternal-child health outcomes [6]. Similarly, vitamin D deficiency has emerged as a widespread health concern in the Indian subcontinent despite abundant ambient sunlight; a recent systematic review reported a pooled prevalence of 66.4% (38,762 participants) among apparently healthy Indian children and adolescents, with comparably high estimates in adult populations [7]. Vitamin B12 deficiency is increasingly recognized as a highly prevalent condition, particularly among vegetarian populations, and contributes to neurological and hematological complications [8].

The coexistence of nutritional deficiencies with overweight and obesity represents a complex public health paradox, in which energy-dense but nutrient-poor diets sustain micronutrient inadequacy alongside excess weight [9]. This pattern is of particular concern because it contributes to the rising burden of non-communicable diseases such as diabetes, cardiovascular disease, and metabolic syndrome [10]. Gender disparities in nutritional status also persist in India, with women experiencing disproportionately higher rates of anemia and micronutrient deficiencies as a result of physiological demands, dietary practices, and sociocultural factors [11].

Family medicine and primary care settings serve as crucial entry points for identifying and managing nutritional deficiencies in the community. The Centre for Nutritional Health at the Department of Community and Family Medicine, All India Institute of Medical Sciences (AIIMS), Mangalagiri, was established to address this need by providing specialized nutritional services, including comprehensive nutritional assessment, individualized nutrition counseling, and diagnosis of key nutritional deficiencies. Systematic assessment of nutritional status in such specialized settings can facilitate early detection, appropriate intervention, and monitoring of treatment outcomes; in an earlier retrospective adolescent cohort from the same center, our group documented a high burden of post-COVID-19 nutritional deficiencies in this catchment [12].

Community surveys such as NFHS-5 provide indispensable national prevalence estimates but rarely capture the clustering of multiple biochemically confirmed micronutrient deficiencies with anthropometric overnutrition within the same individual, and they do not characterize the population that actually presents for nutritional care in primary settings. A clinic-based sample from a dedicated Centre for Nutritional Health therefore offers complementary, practice-oriented evidence: it describes the real-world case-mix that family physicians must manage, quantifies the burden of concurrent deficiencies against a uniform biochemical and anthropometric dataset, and informs the design of pragmatic screening bundles and referral pathways for primary care. Despite this rationale, comprehensive data on the prevalence and patterns of multiple nutritional deficiencies among patients attending nutrition centers in Andhra Pradesh, South India, remain limited.

The primary objective of this study was to estimate the period prevalence of anemia, vitamin D deficiency, vitamin B12 deficiency, and malnutrition (both undernutrition and overnutrition) among adult patients attending the Centre for Nutritional Health, Department of Community and Family Medicine, AIIMS, Mangalagiri, between June 2023 and December 2025. The secondary objectives were to (1) characterize gender-based differences in hematological, biochemical, and anthropometric parameters; (2) describe the age-stratified distribution of each deficiency; (3) quantify the burden of multiple concurrent deficiencies and identify the most frequently co-occurring patterns; and (4) identify independent demographic and anthropometric predictors of vitamin D deficiency.

## Materials and methods

Study design and setting

This was a cross-sectional study based on retrospective data of de-identified clinical records, conducted at the center for Nutritional Health, Department of Community and Family Medicine, AIIMS, Mangalagiri, for patients who attended the center between June 2023 and December 2025. The Centre for Nutritional Health is a specialized service that provides comprehensive nutritional assessment, dietary counseling, and management of nutritional deficiencies for patients from the surrounding community. AIIMS, Mangalagiri is a tertiary-care referral center catering to the healthcare needs of the population of Guntur District, Andhra Pradesh, and the neighboring districts.

Study population

A total of 2,303 patients who attended the Centre for Nutritional Health during the study period were included. Patients with incomplete demographic information in their clinical records were excluded from the analysis.

Sampling approach and sample size justification

A complete enumeration (census) approach was used rather than probability sampling. All patients who attended the center during the defined period and whose clinical records were available and sufficiently complete for analysis were included; no records were excluded on a sampling basis. This yielded a final analytical sample of 2,303 patients.

Because the study included the entire eligible population seen at the center during the defined period, an a priori sample size calculation was not applicable; however, a post-hoc precision analysis was performed to demonstrate that the sample was sufficient to estimate prevalence with acceptable precision. The standard formula for a single proportion was used:

$n = \frac{Z^{2} \cdot p \cdot (1 - p)}{d^{2}}$

where *Z* = 1.96 for a 95% confidence level, *p* = observed prevalence, and *d* = absolute margin of error. For reference, an a priori calculation assuming the most conservative expected prevalence of 50% and an absolute precision of ±5% at 95% confidence would have required a minimum of 385 participants, a threshold exceeded approximately six-fold in the overall sample and exceeded in every individual biochemical subsample. All 95% confidence intervals (CIs) around the prevalence estimates were narrower than ±4%, indicating adequate precision for prevalence estimation in this catchment population.

Data collection

Clinical and demographic data were extracted from existing patient records at the center by the investigators using a standardized data-extraction proforma. Extracted variables included age, sex, anthropometric measurements (weight in kg, height in cm), and available biochemical parameters.

Complete data were not available for every variable in every record, reflecting the real-world heterogeneity of routine clinical documentation. Weight was documented in 1,600 (69.5%), height in 1,595 (69.3%), and body mass index (BMI) - calculable only when both weight and height were available and biologically plausible (weight 20-200 kg; height 100-200 cm) - in 1,590 (69.0%). Five records had weight recorded, but no height or vice versa, and an additional five BMI values were excluded after range checking because one of the constituent measurements fell outside biologically plausible bounds and was judged to represent a transcription error. No imputation of missing values was performed; each analysis used the complete-case denominator, and the denominator for each parameter is stated alongside every numerical estimate in the tables and the text.

Biochemical investigations at the center were ordered by the treating physician based on clinical indication. Universal screening of all the patients was not done because of cost and logistics. The need for biochemical tests was first assessed by Junior Residents, followed by Senior Residents, and the final decision was made by the Centre in Charge. Consequently, results were available only for patients who underwent each test: hemoglobin in 1,610 (69.9%), serum 25-hydroxyvitamin D (25(OH)D) in 1,105 (48.0%), and serum vitamin B12 in 596 (25.9%) of the 2,303 patients. These tested subsets may therefore differ systematically from the full cohort, being enriched for symptomatic or higher-risk individuals; prevalence estimates should be interpreted as conditional on having been tested rather than as unbiased estimates for the full center population or source community. The likely direction of this bias (upward) is addressed formally in the Limitations. A 24-hour dietary recall was administered by a trained counselor but was documented for only 44 (1.9%) patients.

Definitions and criteria

Nutritional deficiencies and malnutrition were classified using standardized criteria.

Anemia

Classified according to World Health Organization (WHO) criteria based on hemoglobin concentration. Normal was defined as ≥13.0 g/dL for males and ≥12.0 g/dL for non-pregnant females. Anemia was categorized by severity as mild (11.0-12.9 g/dL for males; 11.0-11.9 g/dL for non-pregnant females), moderate (8.0-10.9 g/dL for both sexes), and severe (<8.0 g/dL for both sexes) [13].

Vitamin D Status

Serum 25(OH)D levels were categorized as sufficient (≥30 ng/mL), insufficient (20-29 ng/mL), or deficient (<20 ng/mL) according to the Endocrine Society guidelines [14].

Vitamin B12 Status

Classified as normal (≥300 pg/mL), borderline (200-299 pg/mL), or deficient (<200 pg/mL) based on established laboratory reference ranges [15].

Body Mass Index

Calculated as weight (kg) divided by height squared (m²). Asian-specific BMI cutoffs were applied: underweight (<18.5 kg/m²), normal (18.5-22.9 kg/m²), overweight (23.0-24.9 kg/m²), and obese (≥25 kg/m²) [16].

Dietary Intake

Twenty-four-hour dietary recall data were available for only a small convenience subset (n = 24-44; 1.4-1.9% of the cohort); median (interquartile range, IQR) energy, protein, and iron intake were calculated and compared between genders using the Mann. 

Statistical analysis

All statistical analyses were performed in Python v3.11 (Python Software Foundation, Wilmington, DE) using pandas v2.1 and NumPy v1.26 (data management; open-source libraries maintained by the pandas and NumPy development teams under NumFOCUS, Austin, TX); SciPy.stats v1.11 (chi-square, Mann-Whitney U, Shapiro-Wilk, and Spearman rank correlation; open-source libraries maintained by the SciPy development team under NumFOCUS); statsmodels v0.14 (binary logistic regression, variance inflation factor (VIF), and Hosmer-Lemeshow goodness-of-fit; an open-source library maintained by the statsmodels development team under NumFOCUS); and matplotlib v3.8 with seaborn v0.13 for visualization (open-source libraries maintained by the Matplotlib and Seaborn development teams under NumFOCUS). Continuous variables were summarized as mean ± SD or median (IQR) according to distribution, and categorical variables as frequencies and percentages. Chi-square tests were used for categorical comparisons, and odds ratios (ORs) with 95% confidence intervals quantified the strength of association between gender and each nutritional deficiency.

The distribution of each continuous variable (age, hemoglobin, 25(OH)D, vitamin B12, BMI, and dietary energy, protein, and iron intake) was assessed using the Shapiro-Wilk test and by visual inspection of histograms and Q-Q plots. For variables with non-normal distributions, non-parametric methods were used throughout - the Mann-Whitney U test for between-group comparisons of continuous variables, and Spearman's rank correlation coefficient (ρ) for bivariate associations.

Multivariable binary logistic regression was performed to identify independent predictors of vitamin D deficiency (<20 ng/mL). For each bivariate or multivariable analysis, only records with non-missing values for all constituent variables were included. Covariates entered into the multivariable model (age, sex, BMI, and anemia status) were pre-specified a priori on the basis of biological plausibility and prior epidemiological evidence. Age was included because cutaneous vitamin D₃ synthesis and renal 1α-hydroxylase activity both decline with advancing age. Sex was included as a well-characterized predictor of vitamin D status in Indian populations, reflecting differential clothing coverage, outdoor occupational exposure, and pregnancy/lactational demands. BMI was included because vitamin D is fat-soluble and is sequestered in adipose tissue, lowering circulating 25(OH)D per unit cutaneous synthesis. Anemia status was included in view of the bidirectional cross-talk between vitamin D and iron homeostasis - 25(OH)D suppresses hepcidin and modulates erythropoietin responsiveness, while iron-deficiency anemia may impair hepatic 25-hydroxylation of cholecalciferol - and to test whether the sex-vitamin D association was confounded by hematological status. Dietary pattern (energy, protein, and iron intake) was not entered into the model because 24-hour recall data were available for only 44 (1.9%) of the sample, precluding stable multivariable modeling.

Model fit was evaluated using the Hosmer-Lemeshow goodness-of-fit test together with McFadden, Cox & Snell, and Nagelkerke pseudo-R² values, and multicollinearity was assessed using VIF and tolerance (VIF > 5 or tolerance < 0.2 regarded as problematic). A two-sided P < 0.05 was considered statistically significant throughout.

## Results

Baseline characteristics

Table 1 shows that the study included 2,303 patients, with a female predominance of 1681 (73.0%). The mean age of participants was 42.4 ± 13.9 years. The majority were in the 30-44-year age group (879, 38.2%). Using Asian-specific BMI criteria, 994 (62.5%) patients were obese. Among the 1,610 (69.9%) patients in whom it was measured, the mean hemoglobin concentration was 11.08 ± 2.13 g/dL.

**Table 1:** Baseline characteristics of study participants (n = 2,303). BMI, body mass index; IQR, interquartile range; SD, standard deviation

Characteristic	Frequency *n* (%)/Mean ± SD (Median)
Gender (*n *= 2,303)	
Male	622 (27.0%)
Female	1,681 (73.0%)
Age (years) (*n *= 2,303)	
Mean ± SD	42.4 ± 13.9
Median (IQR)	42 (32-52)
Range	11-87
Age categories (*n *= 2,303)	
<18 years	73 (3.2%)
18-29 years	362 (15.7%)
30-44 years	879 (38.2%)
45-59 years	703 (30.5%)
≥60 years	279 (12.1%)
Anthropometric measurements	
Weight (kg), mean ± SD	66.1 ± 13.7 (*n *= 1,600, 69.4%)
Height (cm), median (IQR)	156.0 (152.0-162.0) (*n *= 1,595, 69.2%)
BMI (kg/m²), median (IQR)	26.40 (23.46-29.52) (*n *= 1,590, 69.0%)
BMI categories (Asian criteria) (*n *= 1,590)	
Underweight (BMI < 18.5 kg/m²)	88 (5.5%)
Normal (BMI 18.5-22.9 kg/m²)	262 (16.5%)
Overweight (BMI 23.0-24.9 kg/m²)	246 (15.5%)
Obese (BMI ≥25 kg/m²)	994 (62.5%)
Hemoglobin (g/dL)	
Mean ± SD	11.08 ± 2.13 (*n *= 1,610, 69.9%)

Summary of 24-hour dietary recall

Twenty-four-hour dietary recall data, collected by a trained dietitian, were available for only a small convenience subset of the cohort: 44 (1.9%) patients for energy intake, 33 (1.4%) for protein intake, and 24 (1.0%) for iron intake. In this subset, the median energy intake was 1,585 kcal/day (IQR, 1,371-1,713), the median protein intake was 10.0 g/day (IQR, 8.0-31.3), and the median iron intake was 2.45 mg/day (IQR, 2.00-3.20). Gender-stratified comparison using the Mann-Whitney U test revealed no statistically significant differences between males and females for any of the three dietary variables (all *P* > 0.05; Table 2).

**Table 2:** Descriptive summary of 24-hour dietary recall. *Percentage of the total study cohort (*N* = 2,303). †Percentage of dietary-recall subgroup for the corresponding parameter (*n* = 44 for energy, *n* = 33 for protein, *n* = 24 for iron). IQR, interquartile range; U, Mann-Whitney U statistic

Dietary parameter	Group	*n* (% of cohort/% of subgroup)*	Median (IQR)	Gender comparison (Mann-Whitney U; *P*-value)
Energy intake (kcal/day)	Overall	44 (1.9%)*	1,585 (1,371-1,713)	*U* = 228; *P* = 0.804
	Male	15 (34.1%)†	1,630 (1,202-1,715)	
	Female	29 (65.9%)†	1,550 (1,409-1,670)	
Protein intake (g/day)	Overall	33 (1.4%)*	10.0 (8.0-31.3)	*U* = 165; *P* = 0.202
	Male	13 (39.4%)†	25.0 (10.0-31.3)	
	Female	20 (60.6%)†	9.0 (7.85-32.0)	
Iron intake (mg/day)	Overall	24 (1.0%)*	2.45 (2.00-3.20)	*U* = 81; *P* = 0.075
	Male	6 (25.0%)†	3.50 (2.55-8.50)	
	Female	18 (75.0%)†	2.25 (2.00-3.00)	

Prevalence of nutritional deficiencies

The prevalence of nutritional deficiencies was strikingly high in the study population (Table 3). Among the 1,610 (69.9%) patients who underwent hemoglobin testing, anemia was identified in 1,195 (74.2%); of these, 431 (26.8%) had mild, 675 (41.9%) had moderate, and 89 (5.5%) had severe anemia. Vitamin D status was the most pervasive biochemical abnormality in the cohort: of the 1,105 (48.0%) patients in whom 25(OH)D was measured, 1,059 (95.8%) had suboptimal levels (<30 ng/mL), comprising 658 (59.5%) who were frankly deficient (<20 ng/mL) and 401 (36.3%) in the insufficient range (20-29 ng/mL); only 46 (4.2%) met the sufficiency threshold of ≥30 ng/mL. Vitamin B12 deficiency was identified in 250 (41.9%) of the 596 (25.9%) patients. By Asian-specific BMI criteria, 1,240 (78.0%) patients were overweight or obese, and 88 (5.5%) were underweight. The detailed distribution of nutritional deficiencies is presented in Table 3.

**Table 3:** Prevalence of nutritional deficiencies. BMI, body mass index; CI, confidence interval; WHO, World Health Organization

Nutritional parameter	Prevalence (% of tested)
Malnutrition (BMI-based)	
Underweight (BMI < 18.5 kg/m²)	88 (5.5%)
Normal (BMI 18.5-22.9 kg/m²)	262 (16.5%)
Overweight (BMI 23-24.9 kg/m²)	246 (15.5%)
Obese (BMI ≥ 25 kg/m²)	994 (62.5%)
Anemia status (WHO criteria)	
Normal	415 (25.8%)
Any anemia (combined)	1,195 (74.2%)
Mild anemia	431 (26.8%)
Moderate anemia	675 (41.9%)
Severe anemia	89 (5.5%)
Vitamin D status (ng/mL)	
Sufficient (≥30)	46 (4.2%)
Insufficient (20-29)	401 (36.3%)
Deficient (<20)	658 (59.5%)
Vitamin B12 status (pg/mL)	
Normal (≥300)	211 (35.4%)
Borderline (200-299)	135 (22.7%)
Deficient (<200)	250 (41.9%)

Gender-based comparison of nutritional parameters

Statistically significant gender disparities were observed across multiple demographic, hematological, and biochemical parameters (Table 4). On average, men were older than women (mean age, 46.5 ± 14.8 vs. 40.9 ± 13.3 years; *P* < 0.001).

**Table 4:** Gender-based comparison of nutritional parameters. BMI, body mass index; CI, confidence interval; IQR, interquartile range; SD, standard deviation; OR, odds ratio

Parameter	Male	Female	Test statistic/Effect size	*P*-value
Age (years), mean ± SD	46.5 ± 14.8 (*n* = 620)	40.9 ± 13.3 (*n* = 1,676)	Mann-Whitney U	<0.001
BMI (kg/m²), median (IQR)	26.67 (23.79-28.91) (*n* = 417)	26.25 (23.42-29.83) (*n* = 1,173)	Mann-Whitney U	0.611
BMI categories	*n* = 417 (26.2%)	*n* = 1,173 (73.8%)	χ² = 3.41; df = 3	0.332
Underweight	17 (4.1%)	71 (6.1%)		
Normal	63 (15.1%)	199 (17.0%)		
Overweight	66 (15.8%)	180 (15.3%)		
Obese	271 (65.0%)	723 (61.6%)		
Overweight/Obese vs. normal	337 (80.8%) vs. 63 (15.8%)	903 (81.9%) vs. 199 (18.1%)	χ² = 0.93; df = 1; OR = 0.85 (95% CI, 0.62-1.16)	0.335
Hemoglobin (g/dL), mean ± SD	12.74 ± 2.41 (*n* = 351)	10.62 ± 1.79 (*n* = 1,259)	Mann-Whitney U	<0.001
Anemia	*n* = 351 (21.8%)	*n* = 1,259 (78.2%)	χ² = 146.06; df = 3	<0.001
Normal	166 (47.3%)	249 (19.8%)		
Mild	108 (30.8%)	323 (25.7%)		
Moderate	72 (20.5%)	603 (47.9%)		
Severe	5 (1.4%)	84 (6.7%)		
Any anemia vs. normal	185 (52.7%) vs. 166 (47.3%)	1,010 (80.2%) vs. 249 (19.8%)	χ² = 107.19; df = 1; OR = 3.64 (95% CI, 2.83-4.68)	<0.001
Vitamin D (ng/mL), median (IQR)	19.3 (15.7-23.0) (*n* = 385)	18.0 (13.7-22.1) (*n* = 720)	Mann-Whitney U	<0.001
Vitamin D status	*n* = 385 (34.8%)	*n* = 720 (65.2%)	χ² = 11.64; df = 2	0.003
Sufficient (≥30 ng/mL)	24 (6.2%)	22 (3.1%)		
Insufficient (20-29 ng/mL)	154 (40.0%)	247 (34.3%)		
Deficient (<20 ng/mL)	207 (53.8%)	451 (62.6%)		
Insufficient/Deficient (<30) vs. Sufficient (≥30)	361 (93.8%) vs. 24 (6.2%)	698 (96.9%) vs. 22 (3.1%)	χ² = 5.58; df = 1; OR = 2.11 (95% CI, 1.17-3.81)	0.018
Vitamin B12 (pg/mL), median (IQR)	200.5 (148.25-322.75) (*n* = 221)	254.0 (170.0-397.0) (*n* = 375)	Mann-Whitney U	0.006
Vitamin B12 status	*n* = 221 (37.1%)	*n* = 375 (62.9%)	χ² = 9.91; df = 2	0.007
Normal (≥300 pg/mL)	62 (28.1%)	149 (39.7%)		
Borderline (200-299 pg/mL)	50 (22.6%)	85 (22.7%)		
Deficient (<200 pg/mL)	109 (49.3%)	141 (37.6%)		
Deficient (<200) vs. Normal/Borderline (≥200)	109 (49.3%) vs. 112 (50.7%)	141 (37.6%) vs. 234 (62.4%)	χ² = 7.37; df = 1; OR = 0.62 (95% CI, 0.44-0.87)	0.007

The hematological burden of disease was strongly female-predominant. Mean hemoglobin was substantially lower in women (10.62 ± 1.79 g/dL) than in men (12.74 ± 2.41 g/dL), and the categorical distribution of anemia severity differed significantly between genders (χ² = 146.06; df = 3; *P* < 0.001): only 249 (19.8%) women had normal hemoglobin levels compared with 166 (47.3%) men, and moderate-to-severe anemia was present in 687 (54.6%) women vs. 77 (21.9%) men. On the dichotomized comparison (any anemia vs. normal), women had 3.64 times higher odds of being anemic than men (OR, 3.64; 95% CI, 2.83-4.68; *P* < 0.001).

Vitamin D status showed a similar female-predominant pattern. The median 25(OH)D level was lower in women (18.0 ng/mL; IQR, 13.7-22.1) than in men (19.3 ng/mL; IQR, 15.7-23.0), and the categorical distribution differed significantly between genders (χ² = 11.64; df = 2; *P* = 0.003). Frank deficiency (<20 ng/mL) was present in 451 (62.6%) women and 207 (53.8%) men, whereas only 22 (3.1%) women and 24 (6.2%) men met the sufficiency threshold (≥30 ng/mL). On the dichotomized comparison (insufficient or deficient vs. sufficient), women had more than twice the odds of suboptimal vitamin D status compared with men (OR, 2.11; 95% CI, 1.17-3.81; *P* = 0.018).

The pattern for vitamin B12 ran in the opposite direction. Median serum vitamin B12 was lower in men (200.5 pg/mL; IQR, 148.25-322.75) than in women (254.0 pg/mL; IQR, 170.0-397.0), and the categorical distribution differed significantly between genders (χ² = 9.91; df = 2; *P* = 0.007). Deficiency (<200 pg/mL) was noted in 109 (49.3%) men and 141 (37.6%) women; on the dichotomized comparison (deficient vs. normal or borderline), women had 38% lower odds of vitamin B12 deficiency than men (OR, 0.62; 95% CI, 0.44-0.87; *P* = 0.007). 

By contrast, no significant gender differences were observed in median BMI (26.67 vs. 26.25 kg/m²; *P* = 0.611), in the four-level categorical BMI distribution (χ² = 3.41; df = 3; *P* = 0.332), or in the dichotomized comparison restricted to normal versus overweight/obese with underweight participants excluded (OR, 0.85; 95% CI, 0.62-1.16; *P* = 0.335), indicating that overweight and obesity were similarly prevalent in men and women.

Predictors of vitamin D deficiency

Multivariable logistic regression was performed to identify independent predictors of vitamin D deficiency among the 727 (31.6%) patients with complete data for all covariates (Table 5). After adjustment for age, gender, BMI, and anemia status, female gender (adjusted OR (aOR), 1.60; 95% CI, 1.16-2.21; *P* = 0.004) and younger age (aOR, 0.98 per year; 95% CI, 0.97-0.99; *P* = 0.001) emerged as significant independent predictors of vitamin D deficiency. 

**Table 5:** Multivariable logistic regression: predictors of vitamin D deficiency (<20 ng/mL), n = 727. *Statistically significant (*P *< 0.05). Model fit, Hosmer-Lemeshow χ²=7.39, *P *= 0.495; McFadden pseudo *R*² = 0.023; Nagelkerke pseudo *R*² = 0.042; Cox & Snell pseudo *R*² = 0.031; AIC = 973.9. Multicollinearity: all VIF values 1.04-1.09 (tolerance 0.92-0.97). OR, odds ratio; CI, confidence interval; BMI, body mass index; VIF, variance inflation factor; AIC, Akaike information criterion

Variable	Adjusted OR	95% CI	*P*-value
Female gender	1.6	1.16-2.21	0.004*
Age (per year increase)	0.98	0.97-0.99	0.001*
BMI (per kg/m²)	0.99	0.96-1.03	0.758
Anemia (yes vs. no)	0.83	0.59-1.17	0.299

Age-wise distribution of nutritional deficiencies

The prevalence of anemia followed a bimodal pattern (*P* < 0.001), peaking in adolescents (52, 81.2%) and in adults aged 30-44 years (524, 80.0%), and reaching its nadir in those aged 45-59 years (303, 67.0%), a distribution consistent with the contributions of growth-related and reproductive-age iron demand. Vitamin D deficiency was widely prevalent across all age groups, ranging from 74 (49.3%) in adults aged ≥60 years to 117 (68.0%) in young adults aged 18-29 years, and did not differ significantly by age (χ² = 13.31; *P* = 0.102), indicating a population-wide rather than an age-concentrated phenomenon and reinforcing the near-universal suboptimal 25(OH)D status (1,059, 95.8%) observed in the cohort. Among adolescents (<18 years), vitamin D deficiency was identified in 12 (60.0%); however, this estimate was derived from a small denominator and is too imprecise to be interpreted as a stable adolescent prevalence. Vitamin B12 deficiency varied significantly by age (χ² = 22.28; *P* = 0.004), with the highest prevalence in young adults aged 18-29 years (51, 47.2%) and a modest decline through middle and older age, suggesting an early-adult predominance. Overweight or obesity (BMI ≥ 23 kg/m²) demonstrated the strongest age dependence (χ² = 315.29; *P* < 0.001), rising sharply from 17 (27.9%) in adolescents to 158 (62.5%) in young adults and then plateauing from the fourth decade onward (30-44 years: 516 (82.0%); 45-59 years: 395 (84.9%); ≥60 years: 150 (84.3%)), rendering overnutrition effectively normative by middle age while micronutrient deficiencies remain widespread, underscoring the cohort's *double burden* of disease (Table 6).

**Table 6:** Age-wise distribution of nutritional deficiencies. BMI, body mass index

Nutritional deficiency	Prevalence, *n* (%)	Sample size (*n*)	Statistical test
Anemia prevalence			
<18 years	52 (81.2%)	64	*χ*² = 43.55, *P *< 0.001
18-29 years	193 (72.6%)	266	
30-44 years	524 (80.0%)	655	
45-59 years	303 (67.0%)	452	
≥60 years	122 (70.9%)	172	
Vitamin D deficiency (<20 ng/mL)			
<18 years	12 (60.0%)	20	*χ*² = 13.31, *P *= 0.102
18-29 years	117 (68.0%)	172	
30-44 years	237 (60.6%)	391	
45-59 years	214 (58.2%)	368	
≥60 years	74 (49.3%)	150	
Vitamin B12 deficiency (<200 pg/mL)			
<18 years	4 (40.0%)	10	χ² = 22.28, *P* = 0.004
18-29 years	51 (47.2%)	108	
30-44 years	92 (43.2%)	213	
45-59 years	72 (39.3%)	183	
≥60 years	30 (37.5%)	80	
Overweight/Obesity (BMI ≥23 kg/m^2^)			
<18 years	17 (27.9%)	61	*χ*² = 315.29, *P *< 0.001
18-29 years	158 (62.5%)	253	
30-44 years	516 (82.0%)	629	
45-59 years	395 (84.9%)	465	
≥60 years	150 (84.3%)	178	

Correlations between nutritional parameters

**Figure 1:**
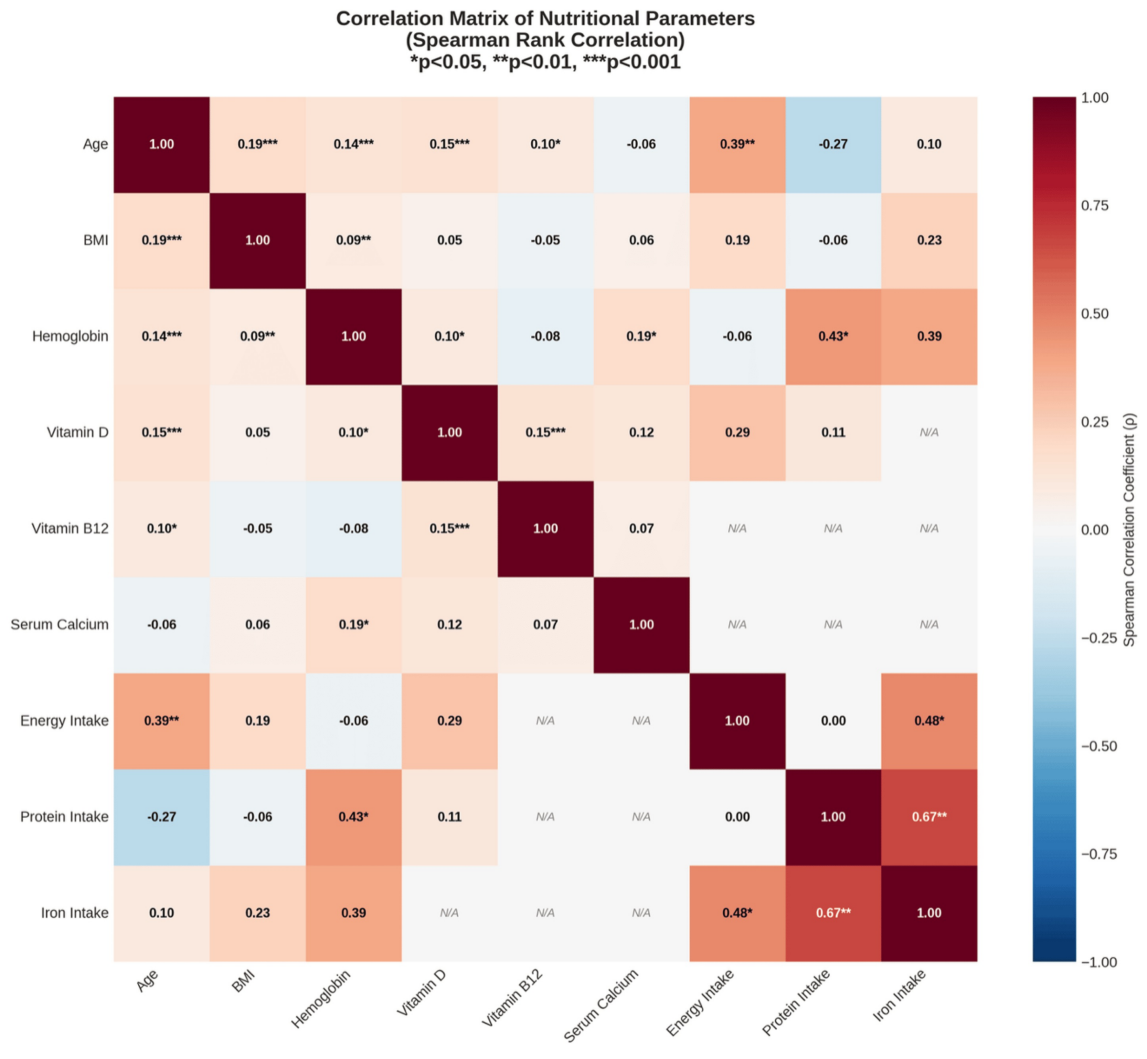
Correlation matrix of nutritional parameters. Image generated using matplotlib v3.8 with seaborn v0.13 (open-source libraries maintained by the Matplotlib and Seaborn development teams under NumFOCUS, Austin, TX) within the Jupyter Notebook environment in Anaconda Navigator (Anaconda, Inc., Austin, TX).

Spearman rank correlation analysis revealed several significant associations among the nutritional parameters (Figure 1). Age demonstrated weak but statistically significant positive correlations with BMI (ρ = 0.19; *P* < 0.001), hemoglobin (ρ = 0.14; *P* < 0.001), 25(OH)D (ρ = 0.15; *P* < 0.001), and vitamin B12 (ρ = 0.10; *P* < 0.05). The strongest age-related association was observed with energy intake (ρ = 0.39; *P* < 0.001); however, this estimate should be interpreted cautiously, as dietary intake data were available only for a small sample. Among biochemical parameters, hemoglobin showed a moderate positive correlation with protein intake (ρ = 0.43; *P* < 0.05), although this estimate was constrained by the small dietary subset, and weak positive correlations with 25(OH)D (ρ = 0.10; *P* < 0.05) and serum calcium (ρ = 0.19; *P* < 0.05). 25(OH)D and vitamin B12 levels were weakly but significantly correlated (ρ = 0.15; *P* < 0.001). Within the dietary parameters, protein and iron intake showed the strongest association in the entire correlation matrix (ρ = 0.67; *P* < 0.01), followed by energy and iron intake (ρ = 0.48; *P* < 0.05).

Multiple concurrent nutritional deficiencies

**Figure 2:**
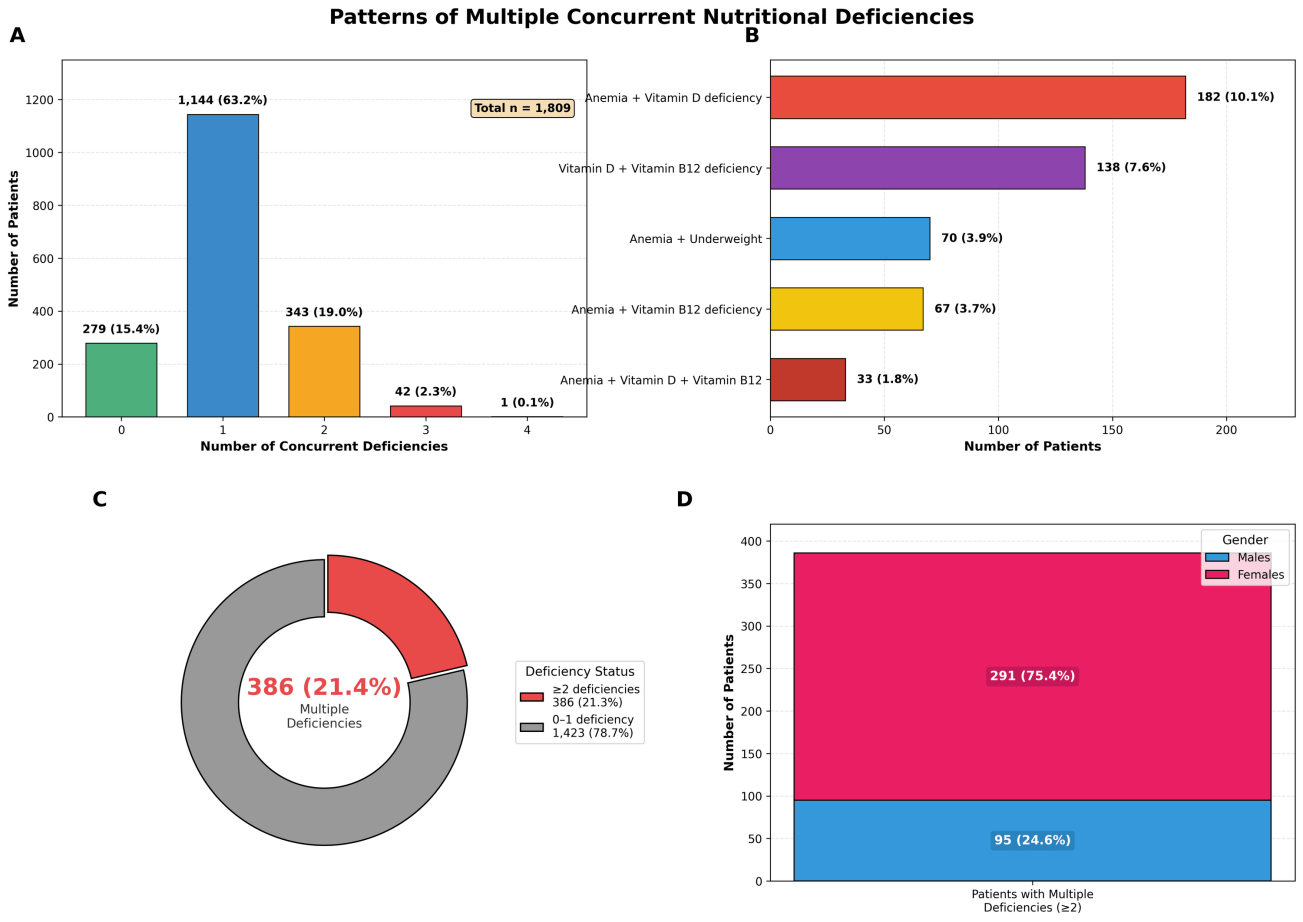
(A-D) Patterns of multiple concurrent nutritional deficiencies. Image generated using matplotlib v3.8 with seaborn v0.13 (open-source libraries maintained by the Matplotlib and Seaborn development teams under NumFOCUS, Austin, TX) within the Jupyter Notebook environment in Anaconda Navigator (Anaconda, Inc., Austin, TX).

Among the 1,809 (78.5%) patients with sufficient biomarker data to determine deficiency status, 1,530 (84.6%) had at least one nutritional deficiency (Figure 2A). The distribution comprised 279 (15.4%) patients with no deficiency, 1,144 (63.2%) with a single deficiency, 343 (19.0%) with two concurrent deficiencies, 42 (2.3%) with three, and 1 (0.1%) with all four. Overall, 386 (21.4%) patients presented with two or more concurrent nutritional deficiencies (Figure 2C). The most common dual-deficiency pattern was anemia combined with vitamin D deficiency, observed in 182 (10.1%) patients (Figure 2B). Triple deficiency comprising anemia, vitamin D deficiency, and vitamin B12 deficiency was identified in 33 (1.8%) patients. Gender stratification revealed a marked disparity in the burden of multiple deficiencies: among the 386 patients with two or more concurrent deficiencies, 291 (75.4%) were female and 95 (24.6%) were male, corresponding to a female-to-male ratio of 3.1:1 (Figure 2D).

## Discussion

In this cross-sectional study of primary-care attendees, multiple micronutrient deficiencies were highly prevalent and frequently coexisted with overweight and obesity. These findings indicate that the contemporary nutritional burden in this catchment is not confined to undernutrition but takes the form of concurrent micronutrient inadequacy and overnutrition - a *double burden* characteristic of the nutrition transition currently underway across India [17].

Burden of anemia

The anemia prevalence in our cohort (1,195, 74.2%) is substantially higher than the national average reported in NFHS-5; however, our cohort comprised adults seeking care at a specialized nutrition center rather than a community-representative sample, and the comparison is therefore subject to Berkson’s bias, which is expected to inflate any prevalence estimate derived from a referral outpatient department relative to its source community. The marked gender disparity, with women having 3.64 times higher odds of anemia than men, aligns with established Indian patterns in which physiological factors (menstruation, pregnancy, and lactation) combine with inadequate dietary iron intake and poor bioavailability from predominantly vegetarian diets [18,19].

The finding that nearly half of the tested individuals had moderate-to-severe anemia is particularly concerning, since these grades are associated with significant functional impairment, reduced work capacity, adverse pregnancy outcomes, and increased mortality risk [20]. The moderate positive correlation between protein intake and hemoglobin level (ρ = 0.43; *P* = 0.014) underscores the importance of dietary protein adequacy, not only for the prevention of protein-energy malnutrition but also for the maintenance of iron status and erythropoiesis [21].

Vitamin D deficiency paradox

The finding that more than half of tested patients were frankly deficient (<20 ng/mL) and an additional approximately one-third insufficient, yielding a near-universal suboptimal 25(OH)D status in Andhra Pradesh, represents a persistent regional and national public health paradox: a population residing at a tropical latitude (≈16° N) with year-round high ultraviolet-B availability that nevertheless carries one of the highest documented burdens of hypovitaminosis D anywhere in the world [22-24]. Our findings replicate, in a primary-care setting, the pattern first described in apparently healthy adults from Tirupati (Andhra Pradesh) by Harinarayan et al., who reported widespread biochemical hypovitaminosis D coexisting with low dietary calcium intake and high phytate consumption despite abundant sunshine [25]; this was extended in their subsequent cross-sectional study of women of reproductive and postmenopausal age, in which vitamin D deficiency was the rule rather than the exception across all age strata [26].

At the national level, Aparna et al. reviewed the convergent evidence base and labeled the phenomenon the "Indian vitamin D paradox," attributing it to a constellation of behavioral, dietary, and environmental factors operating against, rather than alongside, latitude [26]. Consistent with these regional and national data, the determinants invoked in our cohort include cultural and occupational practices favoring sun avoidance, extensive clothing coverage among women, predominantly indoor lifestyles, urbanization and air pollution reducing effective UVB penetration, low dietary calcium and high phytate intake characteristic of South Indian cereal- and pulse-based diets, and constitutive skin pigmentation that lengthens the cutaneous synthesis time required to generate adequate 25(OH)D [25-27]. Within this framework, our cohort's near-universal suboptimal status lies at the upper end of, but is fully consistent with, the regional evidence base.

The low explanatory power of the multivariable logistic regression model (McFadden pseudo *R*² = 0.023; Nagelkerke pseudo *R*² = 0.042) indicates that the adjusted odds ratios are informative as inferential associations but that the model has limited utility as a clinical prediction tool. This highlights the importance of unmeasured determinants - such as sun-exposure behavior, clothing practices, and dietary patterns - and supports a screening-based rather than empirical approach to vitamin D supplementation, particularly given the fat-soluble nature of vitamin D and the potential for toxicity at supraphysiological doses.

Vitamin B12 deficiency

The prevalence of vitamin B12 deficiency in our study is consistent with the well-documented cobalamin inadequacy among predominantly lacto-vegetarian Indian populations, in which animal-source foods contribute only modestly to total intake [28]. The male-predominant pattern, however, runs counter to what shared vegetarian intake would predict and reverses the female-predominant gradient observed for hemoglobin and 25(OH)D in the same participants.

Quantitative South Indian data on male-specific cobalamin intake remain limited: the Comprehensive National Nutrition Survey (CNNS) documented low cobalamin densities across both genders without consistently large male-female intake gaps [29,30], and Indian community studies of B12 status have generally not identified diet alone as the principal driver of inter-individual variation. It is therefore more parsimonious to attribute our finding to non-dietary mechanisms that are plausibly more common in older male OPD attendees: the higher mean age of the male subsample (46.5 vs. 40.9 years), with age-related decline in gastric acid secretion and intrinsic factor availability impairing the liberation of cobalamin from food protein [31]; a substantially higher prevalence of alcohol consumption among adult men than women in Andhra Pradesh according to NFHS-5, with chronic intake impairing absorption and storage [32]; chronic metformin exposure among older male patients with diabetes, dose-dependently associated with cobalamin deficiency [33,34]; and a high background prevalence of Helicobacter pylori infection with male predominance reported in some Indian series, contributing to atrophic gastritis and malabsorption of food-bound cobalamin [35]. These mechanisms are not mutually exclusive and cannot be disentangled in a retrospective dataset; the finding is therefore hypothesis-generating and warrants prospective evaluation with concurrent ascertainment of dietary intake, alcohol use, metformin exposure, and H. pylori status.

Double burden of malnutrition

The coexistence of a high prevalence of overweight or obesity among three-fourths of patients with widespread micronutrient deficiencies exemplifies the double burden of malnutrition characteristic of the nutrition transition [36]. This pattern suggests that patients are consuming energy-dense but nutrient-poor diets, likely dominated by refined grains, added sugars, and limited diversity in fruits, vegetables, and protein sources [37]. The Indian nutrition transition has been marked by an increasing intake of processed foods, sedentary lifestyles, and a decline in the consumption of traditional nutrient-dense foods [38].

The weak positive correlation between BMI and hemoglobin (ρ = 0.09; *P* = 0.002) suggests that, although higher body weight may be modestly associated with better hemoglobin status, this relationship is not strong enough to prevent widespread anemia among overweight and obese individuals. This observation challenges the assumption that adequate energy intake ensures adequate micronutrient status and highlights the concept of *hidden hunger*, in which individuals may be overweight yet micronutrient-deficient [39].

Age-specific nutritional patterns

The age-stratified analysis yielded several insights for targeted intervention. The high anemia prevalence in the 30-44-year age group (8 women of every 10 women) reflects the peak burden among women of reproductive age, in whom menstrual losses, pregnancies, and lactation create substantial iron demands; this finding reinforces the need for systematic screening and supplementation programs targeting women in this age group.

The progressive increase in overweight or obesity prevalence underscores the urgency of early-life interventions. The sharp jump between the 18-29-year and 30-44-year groups suggests that the transition into adulthood and the establishment of sedentary occupational patterns may represent a critical intervention window. The stabilization of overweight or obesity prevalence across all adult age groups ≥30 years indicates that, once established, excess weight tends to persist - reinforcing the importance of prevention strategies aimed at adolescents and young adults before obesity becomes entrenched.

Vitamin B12 deficiency was highest in young adults, among nearly half of patients, possibly reflecting evolving dietary patterns, the increasing adoption of vegetarian diets, or reduced consumption of traditional B12-rich foods among younger generations. This requires targeted dietary counseling for young adults, with emphasis on B12-rich food sources and, where indicated, supplementation.

Gender disparities

The pronounced gender disparities observed in this study reflect persistent inequities in the nutritional status of women in India. The substantially higher burden of anemia and vitamin D deficiency among women has been well documented and reflects a complex interplay of biological, dietary, and sociocultural factors, including intrahousehold food distribution patterns, early marriage and childbearing, inadequate birth spacing, and limited healthcare access [40,41].

These disparities have important implications for maternal and child health, as maternal nutritional deficiencies contribute to adverse pregnancy outcomes, low birth weight, impaired child development, and the intergenerational transmission of malnutrition [42]. Addressing these inequities requires targeted interventions for women of reproductive age, including iron and folic acid supplementation programs, vitamin D supplementation, dietary counseling that emphasizes nutrient-dense foods, and engagement with the underlying social determinants of health [43].

Multiple concurrent deficiencies

The high frequency of concurrent micronutrient deficiencies and the female preponderance among affected patients are consistent with prior Indian community-based data. A community-based study by Kaur et al. among nonpregnant women aged 15-24 years in rural North India found that anemia was present in 256 (60.7%,) while among those tested for micronutrients (*n* = 260), deficiency of ferritin was identified in 170 (65.4%), vitamin B12 in 124 (47.7%), and folate in 48 (18.5%) [44]. Similarly, in a population-based biomarker survey of 979 nonpregnant women of reproductive age (15-40 years) from southern India, Finkelstein et al. reported anemia in 406 (41.5%), vitamin B12 deficiency in 473 (48.3%), and vitamin B12 insufficiency in 727 (74.3%), underscoring the persistent burden of concurrent micronutrient inadequacies in this high-risk demographic [45].

Our concordant positive correlations between 25(OH)D and both vitamin B12 and hemoglobin support the inference that these deficiencies cluster through shared determinants rather than chance, while the non-significant hemoglobin-vitamin B12 association suggests vitamin D occupies a central node connecting iron-related and cobalamin-related axes in this cohort. Plausible upstream drivers in the Indian setting include predominantly vegetarian or lacto-vegetarian diets, which limit bioavailable iron and vitamin B12 [28], restricted cutaneous vitamin D synthesis from cultural sun-avoidance practices and skin pigmentation [25-27], and deeply entrenched intra-household food allocation patterns that disadvantage women, who customarily eat last and receive smaller portions of iron- and protein-rich foods [46,47]. From a practice standpoint, these findings argue for parallel rather than sequential testing of hemoglobin, 25(OH)D, and vitamin B12 in symptomatic women, because identification of one deficiency should prompt evaluation for others.

Role of specialized nutrition centers

The findings from the Centre for Nutritional Health demonstrate the value of specialized nutrition services within primary-care settings. Unlike routine outpatient clinics, in which nutritional assessment may be cursory, the center's focus on comprehensive nutritional evaluation, detailed dietary assessment, and individualized counseling enables identification of the complex deficiency patterns observed in this study.

The Centre for Nutritional Health model - which integrates nutrition counseling, systematic screening for key nutritional deficiencies, and linkage to therapeutic interventions - represents an important approach to addressing the double burden of malnutrition. Such specialized centers can serve as hubs for community nutrition programs, training of healthcare workers, and the development of evidence-based protocols for nutritional management.

The high prevalence of multiple concurrent deficiencies (one-fifth of patients with ≥2 deficiencies) identified through the center's systematic approach emphasizes that comprehensive assessment is essential. Routine clinical encounters might identify anemia but miss coexisting vitamin deficiencies, leading to incomplete treatment. Specialized nutrition services help ensure that all dimensions of nutritional status are evaluated and addressed.

Implications for primary care practice

These findings have important implications for family medicine and primary care practice in India. The Centre for Nutritional Health at AIIMS, Mangalagiri, serves as a model for the integration of specialized nutrition services into primary care, demonstrating the feasibility and value of systematic nutritional assessment. First, routine nutritional screening should be integrated into primary care consultations, particularly for vulnerable populations, including women of reproductive age, pregnant and lactating women, children, and elderly individuals. Second, point-of-care hemoglobin testing should be universally available in primary care to facilitate early detection and management of anemia. Third, empirical supplementation with iron, folic acid, and vitamin B12 may be justified in high-risk populations given the very high prevalence of deficiencies observed [48]. By contrast, vitamin D supplementation should be guided by serum 25(OH)D testing rather than empirical prescription, as vitamin D is fat-soluble and carries the potential for toxicity at supraphysiological doses; unsupervised self-medication without knowledge of baseline levels poses additional risk.

Fourth, dietary counseling should be a core component of primary care consultations, with emphasis on improving dietary diversity, increasing the consumption of protein-rich foods, promoting fortified foods, and ensuring adequate intake of fruits and vegetables. Fifth, addressing the double burden of malnutrition requires simultaneous interventions targeting both undernutrition and overnutrition, including the promotion of balanced diets, physical activity, and prevention of excessive energy intake while ensuring micronutrient adequacy [49].

Strengths and limitations

This study has several strengths. First, the use of complete enumeration rather than probability sampling - every eligible patient seen at the center during the 30-month study period was included - eliminates sampling bias and yields narrow 95% CIs (margin of error ≤ ±4% for every major prevalence estimate), supporting the precision of the reported figures. Second, the large sample size, comprehensive multi-parameter nutritional assessment, application of standardized international deficiency criteria, and examination of gender- and age-stratified patterns, including multiple concurrent deficiencies, allow a granular description of the nutritional landscape in this clinical population. Third, the study characterizes nutritional status among patients actively seeking nutrition-focused healthcare, a population that is poorly represented in community-based surveys but increasingly relevant as nutrition clinics expand in tertiary care.

Several limitations should also be acknowledged. Although the census approach removes sampling bias, the study population was restricted to patients self-referred or clinically referred to a specialized nutrition center at a tertiary institution; the prevalence estimates are therefore conditional on clinical presentation and are not directly generalizable to the source community (Berkson's bias). The subsamples tested for 25(OH)D and vitamin B12 were smaller than the full cohort because biochemical testing was performed on clinical indication rather than universally; post hoc precision nevertheless remained acceptable (±2.9% and ±4.0%, respectively, at 95% CI). The cross-sectional design with retrospective data acquisition precludes inference about temporal relationships or causality. On account of high patient load, difficulty in digitalizing, Twenty-four-hour dietary recall data were available for only a small convenience subset of participants; although gender comparisons using the Mann-Whitney U test showed no statistically significant differences, these analyses are severely underpowered, and the results should not be interpreted as evidence for the absence of a gender gap in dietary intake. Definitive inference will require a prospective study with universal dietary assessment. Detailed data on potential confounders - including socioeconomic status, education, occupation, and habitual dietary patterns - were not collected, limiting our ability to adjust for these variables in the multivariable analyses.

Future research directions

Future research priorities arising from this study include longitudinal evaluations of the impact of nutritional interventions on deficiency prevalence and downstream health outcomes; detailed dietary data assessment using food-frequency questionnaires and dietary-diversity scores; evaluation of nutrition-related knowledge, attitudes, and practices; and assessment of cost-effective strategies for integrating comprehensive nutritional screening into primary care.

## Conclusions

This study found that many patients at the nutrition center had anemia, vitamin D and B12 deficiencies, and were overweight or obese. These problems were more common in women and varied with age. Anemia was most common in women of childbearing age, while overweight and obesity increased sharply during young adulthood and stayed high afterward. These findings suggest that efforts to prevent obesity should focus on young adults, and programs to improve micronutrient levels should prioritize women in their reproductive years.

This study highlights the need for changes in primary care. Instead of testing only when there are signs, a basic set of tests should be done for everyone, including checks for hemoglobin, vitamin D, vitamin B12, and BMI using Asian standards. Women aged 30 to 44 years should be the main focus for treatment with iron, folic acid, vitamin B12, and vitamin D based on blood tests, rather than administering vitamin D without prior testing. Young adults between 18 and 29 years should receive clear advice on healthy living to prevent obesity. Finally, efforts should be made to bring the nutrition center’s approach into local health and wellness centers under Ayushman Bharat.
